# The Effects of a 12-Week Structured Running Programme on the Body Composition and Functional Abilities of Physiotherapy Students

**DOI:** 10.3390/healthcare14162443

**Published:** 2026-08-07

**Authors:** Neven Gladović, Željka Kanižaj Rogina, Nikolina Zaplatić Degač

**Affiliations:** 1 Department of Physiotherapy, University Center Varaždin, University North, 42000 Varaždin, Croatia; ngladovic@unin.hr; 2 Department of Nursing, University Center Varaždin, University North, 42000 Varaždin, Croatia; zkanizaj@unin.hr; 3 Fakulteta za Zdravstvene vede, Univerza v Novem Mestu, 8000 Novo Mesto, Slovenia

**Keywords:** physical activity, students, running, body composition, estimated VO_2_max, physiotherapy, public health

## Abstract

**Highlights:**

**What are the main findings?**

- Participation in a 12-week structured running programme was associated with favourable changes in body composition among physiotherapy students.
- The intervention group showed greater improvements in estimated cardiorespiratory fitness and forward-reach flexibility than the control group.

**What are the implications of the main findings?**

- No significant between-group differences were observed in lower-limb explosive power, suggesting that running alone may not be sufficient to improve this outcome.
- The findings support further evaluation of structured physical activity programmes as a feasible strategy for improving health-related fitness in university settings.

**Abstract:**

**Background/Objectives:** Insufficient physical activity among university students is a significant public health concern associated with unfavorable changes in body composition and reduced cardiorespiratory fitness. This study examined whether participation in a 12-week structured running programme was associated with changes in body composition and selected components of health-related physical fitness in physiotherapy students compared with an inactive control group. **Methods:** A total of 64 physiotherapy students participated in this quasi-experimental study and self-selected into an experimental (*n* = 22) or control group (*n* = 42). The experimental group participated in a 12-week structured running programme comprising two supervised running sessions (Monday and Friday) and one brisk walking session (Wednesday) per week. Although brisk walking was incorporated as one weekly aerobic training session, running represented the principal training modality throughout the intervention, whereas the control group did not participate in organized physical activity. Body composition, estimated maximal oxygen uptake (estimated VO_2_max), forward-reach flexibility, and standing long jump performance were assessed before and after the intervention. The primary analysis was performed using analysis of covariance (ANCOVA), with post-intervention values as dependent variables, study group as the fixed factor, and baseline values together with sex included as covariates. Statistical significance was interpreted after adjustment for multiple comparisons using the Holm procedure. **Results:** After adjustment for baseline values and sex, the intervention group showed significantly greater improvements than the control group in body mass (*p* < 0.001), body fat percentage (*p* < 0.001), visceral fat rating (*p* = 0.002), body mass index (*p* < 0.001), total body water (*p* = 0.047), estimated VO_2_max (*p* = 0.019), and forward-reach flexibility (*p* = 0.002). No statistically significant between-group difference was observed for standing long jump performance (*p* = 0.124). The largest adjusted effect sizes were observed for body mass index and body fat percentage. **Conclusions:** Participation in a 12-week structured running programme was associated with more favorable changes in body composition, estimated cardiorespiratory fitness, total body water, and forward-reach flexibility compared with the control group. These findings suggest that structured running programmes may warrant further evaluation as a potential university-based health-promotion approach.

## 1. Introduction

Insufficient physical activity among university students represents a growing public health concern [1,2]. The transition from secondary education to higher education is frequently accompanied by unhealthy lifestyle modifications, including increased sedentary behaviour, a decline in habitual physical activity, and adverse changes in body composition, such as increases in body mass and adiposity [3]. Although young adults often present with a normal body mass index (BMI), lower levels of physical activity during this developmental stage can negatively affect cardiorespiratory fitness, musculoskeletal flexibility, and overall metabolic health, establishing long-term health risks that persist into adulthood [3,4].

Aerobic exercise, particularly running, is a highly accessible and cost-effective strategy for enhancing cardiorespiratory endurance and optimizing body composition [5]. Regular aerobic training increases total energy expenditure, enhances fatty acid oxidation, and induces cardiovascular adaptations [6,7]. However, despite extensive literature evaluating aerobic exercise in general and clinical populations, evidence regarding structured, low-equipment exercise interventions implemented within higher education settings remains limited [8]. Furthermore, most existing studies focus on either body composition or isolated physical performance metrics. In contrast, comprehensive evaluations that concurrently monitor body composition and multiple components of health-related physical fitness are scarce [9].

Healthcare students (including physiotherapy, nursing, and medical students) represent a particularly important population for health promotion research [10]. As future healthcare professionals, they will be responsible for prescribing therapeutic exercise and promoting active lifestyles to their patients [10,11]. Maintaining personal physical fitness and participating in structured exercise programmes during their university education may not only protect their own physical health, but also enhance their professional credibility and competence as advocates for physical activity [11,12]. Nevertheless, baseline physical activity levels among university students often fall short of international public health recommendations, underscoring the need for feasible, university-based physical activity interventions [10,13].

Accordingly, this study aimed to investigate the effects of a 12-week structured running programme on body composition and health-related physical fitness in previously inactive physiotherapy students compared to an inactive control group. It was hypothesized that participation in the 12-week structured running programme would be associated with significant reductions in adiposity indicators (body mass, body fat percentage, visceral fat rating, and body mass index) and improvements in estimated cardiorespiratory fitness (estimated VO_2_max) and forward-reach flexibility compared with non-participating controls. The primary outcomes were body composition parameters and estimated cardiorespiratory fitness, while lower limb explosive power and forward-reach flexibility were analyzed as secondary outcomes.

## 2. Materials and Methods

### 2.1. Study Design

The study was conducted as a 12-week longitudinal quasi-experimental study with a control group. Measurements were performed at two time points: before the start of the intervention (baseline measurement) and after the completion of the programme (post-intervention measurement). Due to the voluntary enrollment of participants in the intervention, group allocation was non-randomized.

Baseline assessments were performed between 15 and 19 September 2025, prior to the start of the intervention. The first training session of the 12-week structured running programme was conducted on 22 September 2025, while the final training session took place on 12 December 2025. Post-intervention assessments were performed between 15 and 19 December 2025, immediately following completion of the intervention programme. All measurements and intervention procedures were carried out at the facilities of the Department of Physiotherapy, University North, Croatia.

### 2.2. Participants

**Figure 1:**
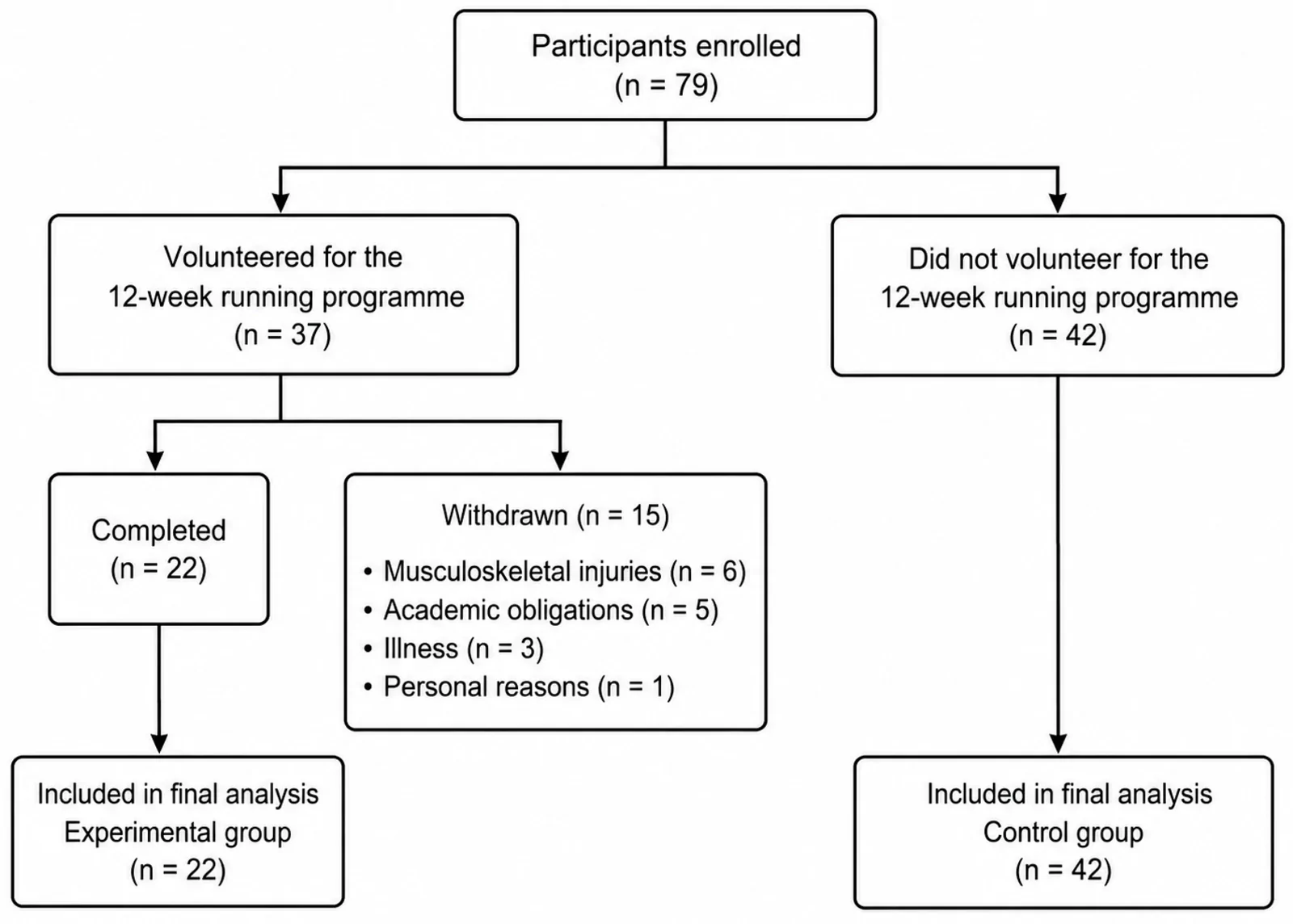
Participant flow diagram illustrating participant recruitment, withdrawals, and the final study sample.

A total of 79 first-year physiotherapy students who had not participated in organized physical activity for at least six months prior to the study were enrolled. Initially, 37 students voluntarily enrolled in the structured running programme. During the intervention, 15 participants withdrew from the study. The reasons for withdrawal included musculoskeletal injuries unrelated to the intervention (*n* = 6; four knee injuries and two cases of low back pain), academic obligations (*n* = 5), illness (*n* = 3), and personal reasons (*n* = 1). According to participant self-reports, none of the reported musculoskeletal injuries occurred during the prescribed running programme or were considered intervention-related. A per-protocol analysis, rather than an intention-to-treat analysis, was performed. Therefore, only participants who completed the intervention and the post-intervention assessments were included in the final statistical analyses (Figure 1).

Consequently, the experimental group consisted of 22 participants who completed the intervention, whereas the control group consisted of 42 students who did not participate in the running programme.

The experimental group consisted of students who voluntarily enrolled in the structured running programme, whereas the control group did not participate in any organized physical activity during the study period. Because participants self-selected into the intervention, allocation was non-randomized, which should be considered when interpreting the findings. Baseline characteristics were compared between the experimental and control groups using the Mann–Whitney U test. No statistically significant differences were observed in any of the analysed variables (*p* > 0.05).

Both groups included seven male participants, while the remaining participants were female. All first-year undergraduate physiotherapy students were informed about the study during lectures within the course Fundamentals of Motor Transformations, during which the health benefits of regular physical activity were presented as part of the course content. Participation in both the study and the running programme was entirely voluntary. Students who expressed an interest in participating were enrolled in the 12-week structured running programme and constituted the intervention group, whereas students who chose not to participate served as the control group. Participation or non-participation in the study had no influence on course requirements or final grades. A formal a priori sample-size calculation was not performed. All eligible first-year physiotherapy students meeting the inclusion criteria during the study period were invited to participate.

Participants were eligible for inclusion if they were first-year physiotherapy students at University North, aged 18–30 years, had not engaged in organized physical activity during the six months preceding the study, were apparently healthy, and provided written informed consent.

Participants were excluded if they had musculoskeletal, cardiovascular, respiratory, or other health conditions contraindicating participation in aerobic exercise, had participated in organized physical activity within the previous six months, failed to complete the baseline or follow-up assessments, or attended fewer than 80% of the prescribed training sessions.

### 2.3. Ethical Considerations

The study was approved by the Ethics Committee for Research Approval of the University North (Class: 641-01/25-01/07; Registration Number: 2137-0336-07-25-1; approval date: 17 June 2025). All participants provided written informed consent before participation, and the study was conducted in accordance with the Declaration of Helsinki.

### 2.4. Running Intervention

The experimental group participated in a 12-week structured running programme comprising two supervised running sessions (Monday and Friday) and one brisk walking session (Wednesday) per week. Although brisk walking was incorporated as one weekly aerobic training session, running represented the principal training modality throughout the intervention. The programme was progressive in nature and combined interval and continuous aerobic exercise, with training duration gradually increasing from approximately 25 min during the initial weeks to 60 min during the final week of the intervention. Maximal heart rate was estimated using the age-predicted formula (220 − age), which is widely used in exercise prescription despite its known individual variability and limited accuracy for estimating true maximal heart rate. The formula was used as a practical method for prescribing training intensity in this field-based intervention. Training intensity zones were prescribed as a percentage of the estimated maximal heart rate. Zone 2 corresponded to 60–75% of HRmax, whereas Zone 3 corresponded to 75–85% of HRmax [8].

Each training session was preceded by a standardized 10 min warm-up consisting of light jogging, dynamic mobility exercises, and running-specific drills (e.g., high knees, heel kicks, and skipping exercises). Following the main part of the training session, participants completed a 5–10 min cool-down period consisting of low-intensity walking and static stretching exercises. Participants were instructed to maintain an exercise intensity corresponding to Zone 3 (75–85% of the estimated HRmax). Most participants monitored exercise intensity using commercially available wearable devices or mobile applications.

Participants without heart rate monitoring devices were instructed to regulate exercise intensity using the Talk Test and maintain a running pace at which comfortable conversation became difficult, which has been shown to correspond closely to moderate-to-vigorous aerobic exercise intensity [14]. Six participants used heart rate monitors or smartwatches, eleven used mobile applications (Strava, https://www.strava.com, accessed 6 August 2026; Garmin Connect, https://connect.garmin.com, accessed 6 August 2026; Apple Health, https://www.apple.com/health, accessed 6 August 2026; Samsung Health, https://www.samsung.com/apps/samsung-health, accessed 6 August 2026), and five regulated exercise intensity using the Talk Test.

The intervention was divided into four progressive phases: an initial phase (Weeks 1–2) consisting of 1–2 min running intervals interspersed with 1.5 min of active recovery (25–35 min total duration); an intermediate phase (Weeks 3–6) consisting of 3–6 min intervals with shorter recovery periods (30–40 min total duration); an advanced phase (Weeks 7–10) consisting of 8–20 min intervals with minimal recovery periods (30–45 min total duration); and a final phase (Weeks 11–12) consisting of continuous running for 40–60 min (Table 1).

The running surface was not standardized because the intervention was designed to reflect real-world exercise conditions; however, most training sessions were performed on a standard outdoor athletics track. Training sessions were performed individually and were not directly supervised by the investigators. Although each session began with a standardized 10 min warm-up and ended with a 5–10 min cool-down, these components were not included in the reported training duration. Participants who missed a scheduled training session were instructed to complete the same session independently within the same week whenever possible, following the prescribed training programme. All training sessions were recorded using digital platforms and mobile applications (e.g., Strava, Garmin Connect, Apple Health, and Samsung Health). Participants uploaded their training records to individual Google Drive folders (Google LLC, Mountain View, CA, USA), and adherence to the prescribed training volume and intensity was monitored after each session by a qualified kinesiologist who reviewed all submitted training data. The intervention consisted of a total of 36 prescribed training sessions. To be eligible for the per-protocol analysis, participants were required to complete at least 80% of the prescribed sessions (≥29 of 36 sessions). All 22 participants who completed the intervention fulfilled this adherence criterion.

### 2.5. Outcome Measures

#### 2.5.1. Body Composition

Body composition was assessed using a multifrequency segmental bioelectrical impedance analyzer (Tanita MC-780MA; Tanita Corporation, Tokyo, Japan). The analyzer uses segmental multifrequency bioelectrical impedance technology to estimate body composition parameters, including body mass, body fat percentage, visceral fat rating, BMI, and total body water. Data acquisition and storage were performed using GMON Health Monitor software (Version 3.4.8; MedServ GmbH, Hamburg, Germany). All measurements were performed under standardized conditions between 08:00 and 10:00 a.m. Participants were measured barefoot and in light clothing and were instructed to refrain from eating or drinking prior to testing. Basic anthropometric data, including age, sex, body height, and body mass, were entered into the system before analysis. The variables analyzed in the present study included body mass, body fat percentage, visceral fat rating, BMI, and total body water.

#### 2.5.2. Functional and Motor Abilities

Explosive power of the lower extremities was assessed using the standing long jump test, which is considered a valid and reliable field method for this purpose [15].

Forward-reach flexibility was measured using the seated forward bend test (straddle position). The participant sits on the floor with legs spread apart by a distance of two foot-lengths. In this position, the participant extends their arms forward, placing the palm of the right hand on the back of the left hand. The examiner places a centimeter tape between the legs so that the 40 cm mark aligns exactly with the imaginary line connecting the heels. After two gentle preliminary bends, the participant lowers themselves into the maximum possible forward bend, which must be held for three seconds [16].

Cardiorespiratory fitness was assessed indirectly using the 20 m shuttle run (Beep) test. This field test has demonstrated good validity and reliability for estimating cardiorespiratory fitness in population-based studies [17]. Estimated maximal oxygen uptake (estimated VO_2_max), expressed in mL·kg^−1^·min^−1^, was determined using the normative conversion table developed by Léger et al. (1988), based on the participant’s age and the final stage completed during the test [18].

### 2.6. Statistical Analysis

Statistical analyses were performed using Spotfire Statistica software (Version 14.3.0.6; Cloud Software Group, Inc., Palo Alto, CA, USA). The normality of data distribution was assessed using the Shapiro–Wilk test. Baseline demographic characteristics (sex and age categories) were compared between the experimental and control groups using Fisher’s exact test. Baseline outcome variables were compared between the experimental and control groups using the Mann–Whitney U test. In addition, baseline characteristics of participants who completed the intervention and those who withdrew before study completion were compared using the Mann–Whitney U test. As a sensitivity analysis, unadjusted mean changes (Δ) between the experimental and control groups were compared using Welch’s *t*-test, and effect sizes were expressed as Cohen’s d. The primary analysis of intervention effects was performed using analysis of covariance (ANCOVA), with post-intervention values as dependent variables, study group as the fixed factor, and the corresponding baseline value together with sex included as covariates. Prior to conducting the ANCOVA analyses, model assumptions were evaluated by inspection of residual plots and by testing the homogeneity of regression slopes using the interaction between group and baseline values. No substantial violations of the model assumptions were identified. ANCOVA analyses were performed using participants with complete baseline and post-intervention data for each outcome variable. Age was not included in the final model because the study sample was relatively homogeneous and preliminary model assessment indicated that age was not a statistically significant covariate. Adjusted mean differences with 95% confidence intervals (95% CI) were calculated. Effect sizes were expressed as partial eta squared (η^2^_p_), with values of ≥0.01, ≥0.06, and ≥0.14 interpreted as small, medium, and large effects, respectively. To account for multiple comparisons, *p*-values obtained from the ANCOVA models were adjusted using the Holm procedure. Statistical significance was set at *p* < 0.05.

## 3. Results

### 3.1. Comparison of Participants Who Completed the Intervention and Those Who Withdrew

Baseline age and study outcome variables of participants who completed the intervention were compared with those of participants who withdrew before study completion using the Mann–Whitney U test (Table 2). No statistically significant differences were observed for any of the analyzed variables (all *p* > 0.05), indicating no evidence of selective attrition based on the measured baseline variables.

### 3.2. Baseline Characteristics

Baseline demographic characteristics and outcome variables were compared between the control and experimental groups. Sex and age distributions were assessed using Fisher’s exact test (Table 3), whereas baseline outcome variables were compared using the Mann–Whitney U test (Table 4).

No statistically significant differences were observed between the groups in either sex distribution (*p* = 0.208) or age categories (*p* = 0.521).

No statistically significant between-group differences were observed for any analyzed baseline outcome variable (all *p* > 0.05).

### 3.3. Sensitivity Analysis

Welch’s *t*-test was performed as a sensitivity analysis to compare the unadjusted mean changes (Δ) between the experimental and control groups (Table 5). Statistically significant between-group differences were observed for body mass (*p* < 0.001), body fat percentage (*p* < 0.001), visceral fat rating (*p* = 0.003), BMI (*p* < 0.001), forward-reach flexibility (*p* < 0.001), and estimated VO_2_max (*p* = 0.015). Large effect sizes (Cohen’s d ≥ 0.80) were observed for body mass, body fat percentage, visceral fat rating, BMI, and forward-reach flexibility. The largest effect sizes were observed for body fat percentage (d = 1.28) and BMI (d = 1.27). No statistically significant between-group differences were observed for total body water percentage (*p* = 0.073) or standing long jump performance (*p* = 0.238).

### 3.4. Primary Analysis (ANCOVA)

Table 6 presents the primary analysis of intervention effects using analysis of covariance (ANCOVA), with baseline values and sex included as covariates in the model. Analyses were performed using participants with complete baseline and post-intervention data for each outcome variable. Consequently, the number of participants included in each ANCOVA model differed slightly across outcome variables because not all participants completed every outcome assessment at both measurement time points. Therefore, analyses were performed using complete cases separately for each outcome, and no missing data were imputed. Following adjustment for these covariates, the intervention group demonstrated significantly greater adjusted improvements in body mass, body fat percentage, visceral fat rating, and BMI compared with the control group. Significant between-group differences were also observed for total body water, estimated VO_2_max, and forward-reach flexibility, whereas no statistically significant difference was found for standing long jump performance.

## 4. Discussion

The present study investigated the effects of a 12-week structured running programme on body composition and selected components of health-related physical fitness in physiotherapy students following participation in a 12-week structured running programme. Compared with the control group, participants in the intervention group exhibited more favourable changes in body composition, estimated cardiorespiratory fitness, and forward-reach flexibility, whereas no significant between-group differences were observed in lower-limb explosive power.

The favourable changes in body composition observed after the 12-week intervention are consistent with previous evidence demonstrating the effectiveness of structured aerobic exercise in healthy young adults. In a study involving previously inactive female university students, Lu et al. [5] reported significant improvements in body composition following a 12-week running-based training programme, including reductions in body fat percentage, findings that closely align with the present results. Notably, although the participants in our study had a mean baseline BMI within the normal range, significant reductions were observed in body mass, body fat percentage, visceral fat rating, and BMI, indicating that structured aerobic exercise may improve body composition even in healthy, previously inactive university students. Although Willis et al. [7] investigated overweight adults, the direction of change observed in the present study was similar despite the participants having a normal mean BMI at baseline. Likewise, Bellicha et al. [19], in an overview of systematic reviews and meta-analyses, concluded that structured exercise consistently reduces body weight, total body fat, and visceral adipose tissue across a wide range of intervention studies, providing high-level evidence for the beneficial effects of aerobic exercise on body composition. These observations indicate that the present findings are consistent with the broader evidence supporting aerobic exercise as an effective intervention for improving adiposity-related outcomes. Although the physiological mechanisms underlying aerobic training adaptations have been well described in previous research, these mechanisms were not directly assessed in the present study. Regular aerobic exercise increases total energy expenditure and induces metabolic adaptations, including enhanced mitochondrial oxidative capacity and increased fatty acid oxidation, thereby promoting progressive reductions in adipose tissue. These mechanisms have been comprehensively described by Swift et al. [6] and provide a plausible explanation for the improvements observed in the present study. Furthermore, the large effect sizes recorded for body fat percentage and BMI indicate that the intervention was not only statistically significant but also practically meaningful. Collectively, these findings suggest that participation in a progressive running programme performed three times per week may be associated with favourable changes in body composition among previously inactive physiotherapy students.

The significant improvement in estimated VO_2_max observed following the 12-week structured running programme is consistent with previous evidence demonstrating that regular aerobic exercise effectively enhances cardiorespiratory fitness in previously inactive young adults. A 12-week running-based intervention conducted by Lu et al. [5] similarly reported significant improvements in aerobic capacity among female university students, supporting the effectiveness of structured running programmes in populations comparable to that investigated in the present study. Furthermore, the meta-analysis by Scribbans et al. [9] confirmed that aerobic exercise training consistently improves estimated VO_2_max in healthy young adults across different training intensities and exercise protocols, indicating that the present findings are consistent with the broader body of evidence. The observed improvement in estimated cardiorespiratory fitness may be related to well-established central and peripheral adaptations to endurance training. Regular aerobic exercise increases stroke volume and maximal cardiac output while promoting skeletal muscle capillarization, mitochondrial biogenesis, and oxidative enzyme activity, thereby enhancing oxygen delivery and utilization during exercise. These physiological adaptations, comprehensively described by Bassett and Howley [20], provide a plausible explanation for the increase in estimated VO_2_max observed in the present study. Although these physiological adaptations are well-established responses to aerobic training, they were not directly measured in the present study. Therefore, the observed improvement should be interpreted as an increase in estimated relative cardiorespiratory fitness rather than as direct evidence of specific central or peripheral physiological adaptations.

From a clinical and public health perspective, the improvement in cardiorespiratory fitness is particularly important because cardiorespiratory fitness is recognized as one of the strongest independent predictors of cardiovascular and all-cause mortality. Accordingly, the American Heart Association [12] recommends that cardiorespiratory fitness should be routinely considered in clinical practice as a clinical vital sign. Therefore, the increase in estimated VO_2_max observed in the present study suggests that a structured running programme performed three times per week may provide meaningful health benefits for previously inactive physiotherapy students, extending beyond improvements in physical performance alone. It should be noted that cardiorespiratory fitness was assessed using estimated relative VO_2_max (mL·kg^−1^·min^−1^). Therefore, part of the observed improvement may reflect the reduction in body mass rather than an increase in absolute aerobic capacity.

The significant improvement in forward-reach flexibility observed following the intervention suggests that regular participation in a structured running programme may contribute to enhanced flexibility of the posterior kinetic chain in previously inactive physiotherapy students. Although relatively few intervention studies have specifically examined the effects of running on forward-reach flexibility, the present findings indicate that repeated dynamic movement performed over a prolonged training period may positively influence flexibility-related outcomes. A possible explanation for the observed improvement is that the repetitive cyclic movements performed during running expose the hip, pelvic, and lumbar regions to repeated dynamic loading throughout a large range of motion. Over time, these adaptations may reduce passive muscle stiffness, increase stretch tolerance, and improve functional flexibility. This interpretation is supported by evidence demonstrating that regular exercise interventions can improve joint range of motion even in the absence of dedicated stretching programmes. In particular, Afonso et al. [21] reported that chronic exercise interventions are capable of increasing range of motion through neuromuscular adaptations, suggesting that improvements in flexibility are not exclusively dependent on stretching exercises. Although flexibility was not the primary target of the present intervention, the observed increase in forward-reach flexibility may represent an additional health-related benefit of regular aerobic exercise. Considering the limited evidence regarding the influence of structured running programmes on forward-reach flexibility in healthy young adults, further randomized controlled studies are warranted to clarify the mechanisms underlying these adaptations. In addition, because each training session included dynamic mobility exercises during the warm-up and static stretching during the cool-down, the observed improvement in forward-reach flexibility cannot be attributed exclusively to running.

In contrast to the improvements observed in body composition, cardiorespiratory fitness, and forward-reach flexibility, the present study did not demonstrate a significant improvement in lower-limb explosive power, as assessed by the standing long jump test. This finding is not unexpected, as the intervention was specifically designed to improve aerobic capacity rather than neuromuscular performance. Similarly, Lu et al. [5] reported that although a 12-week running-based programme improved aerobic fitness and body composition in female university students, gains in muscle fitness were smaller than those achieved following functional high-intensity training, highlighting the limited capacity of running alone to enhance explosive performance. The absence of significant improvements in explosive power is consistent with the principle of training specificity, which states that physiological adaptations are specific to the type, intensity, and velocity of the training stimulus. Since the present intervention primarily consisted of moderate-to-vigorous aerobic running, it provided insufficient neuromuscular overload to induce adaptations associated with maximal force production and explosive movement. Behm and Sale [22] demonstrated that improvements in explosive performance require high-velocity, high-force neuromuscular stimuli that differ substantially from those elicited during endurance exercise. These findings suggest that although structured running programmes effectively improve several health-related fitness components, additional resistance or plyometric training would be required to enhance lower-limb explosive power. Therefore, future interventions targeting comprehensive physical fitness in physiotherapy students should consider combining aerobic and neuromuscular training modalities.

The findings of the present study may have practical implications for health promotion within higher education. University students frequently experience a decline in physical activity levels after enrolment, increasing their risk of developing unfavourable lifestyle behaviours that may persist into adulthood. The present findings suggest that structured running programmes may represent a potentially accessible approach for promoting physical activity within university settings. However, their feasibility, acceptability, and implementation costs were not directly evaluated in the present study and should be examined in future research. Such programmes could therefore be considered for incorporation into university health promotion initiatives or extracurricular physical activity programmes. For physiotherapy students in particular, maintaining an adequate level of physical fitness may also be professionally relevant, as future healthcare professionals play an important role in promoting physically active lifestyles and may serve as positive role models for their patients. Consequently, integrating structured physical activity programmes into university education may contribute to both improved student health and the development of positive professional health behaviours.

Despite the encouraging findings, several strengths and limitations of the present study should be acknowledged. The major strengths include the prospective controlled study design, the implementation of a standardized 12-week running programme, and the comprehensive assessment of both body composition and health-related physical fitness variables in previously inactive physiotherapy students. Furthermore, standardized outcome assessment procedures were used, and participants followed a predefined training programme while adherence was monitored through uploaded training records.

Nevertheless, several limitations should be considered when interpreting the findings. First, the quasi-experimental, non-randomized design may have introduced selection bias because participants self-selected into the intervention group. Although no statistically significant baseline differences were observed between the groups, residual confounding due to the non-randomized design cannot be completely excluded.

In addition, 15 of the 37 participants initially enrolled in the intervention group withdrew before study completion, corresponding to an attrition rate of 40.5%. Six withdrawals were attributed to musculoskeletal injuries (four knee injuries and two cases of low back pain), which were reported by participants as unrelated to the prescribed intervention. Nevertheless, because these events occurred during the study period, future supervised trials should prospectively monitor and report adverse events using standardized procedures to further strengthen the evidence regarding the safety profile of structured running programmes. Although no statistically significant baseline differences were observed between completers and non-completers for the measured variables, selective attrition based on unmeasured characteristics cannot be excluded. The per-protocol, complete-case approach may have overestimated the observed programme-associated changes, as participants who did not complete the intervention were excluded and an intention-to-treat analysis was not performed. Second, the unequal sample sizes between the experimental and control groups may have influenced statistical power. Furthermore, the number of participants included in individual analyses varied across outcome measures owing to missing data for specific assessments. This may have reduced the statistical power for certain outcomes and should be considered when interpreting the findings.

Third, body composition was assessed using bioelectrical impedance analysis rather than dual-energy X-ray absorptiometry (DXA), while cardiorespiratory fitness was estimated indirectly using the 20 m shuttle run test instead of direct cardiopulmonary exercise testing.

Finally, dietary intake and physical activity performed outside the intervention were not monitored, and therefore their potential influence on the observed outcomes cannot be excluded. In addition, physical activity performed by participants in the control group during the intervention period was not systematically monitored. Therefore, changes in habitual physical activity among control participants during the study cannot be excluded, which may have influenced the observed between-group differences. Furthermore, heart rate values achieved during individual training sessions were not systematically recorded. Although exercise intensity was prescribed using estimated heart-rate zones and monitored using wearable devices, mobile applications, or the Talk Test, objective verification of the achieved training intensity was not available for all participants. Consequently, the actual mean exercise intensity and time spent within the prescribed heart-rate zones could not be quantified.

In addition, outcome assessors were not formally blinded to group allocation, which may have introduced measurement bias.

A formal a priori sample-size calculation was not performed. All eligible first-year physiotherapy students meeting the inclusion criteria during the study period were invited to participate.

Finally, because the study was conducted at a single university and included only first-year physiotherapy students, the generalizability of the findings to other student populations should be interpreted with caution.

Future studies should employ randomized controlled designs with larger and more balanced samples to strengthen causal inference. The inclusion of objective measures of habitual physical activity, dietary monitoring, and more precise assessment methods, such as dual-energy X-ray absorptiometry for body composition and direct measurement of maximal oxygen uptake, would further improve methodological quality. In addition, future research should investigate the long-term sustainability of the observed adaptations and evaluate whether combining structured running programmes with resistance or neuromuscular training produces greater improvements across multiple components of health-related physical fitness. Such studies would contribute to the development of evidence-based physical activity programmes for university students and provide further guidance for health promotion strategies within higher education.

## 5. Conclusions

The present study demonstrated that participation in a 12-week structured running programme was associated with favourable changes in several components of health-related physical fitness among previously inactive physiotherapy students, particularly body composition, estimated cardiorespiratory fitness, and forward-reach flexibility, whereas no significant improvement was observed in lower-limb explosive power. These findings suggest that a progressive running programme performed three times per week may represent a practical approach to promoting several components of health-related physical fitness in university students.

Nevertheless, the findings should be interpreted with caution owing to the quasi-experimental, non-randomized study design, participant self-selection, relatively high attrition rate, the absence of a formal a priori sample size calculation, and other methodological limitations. In addition, the relatively small intervention group and outcome-specific missing data may have reduced the precision of some adjusted estimates despite the presentation of adjusted mean differences and 95% confidence intervals. Consequently, causal inferences cannot be drawn from the present findings. Future randomized controlled trials with larger and more diverse samples, objective monitoring of habitual physical activity and exercise intensity, dietary assessment, and longer follow-up periods are warranted to confirm these findings and to determine the long-term effectiveness of structured running programmes in university populations.

## Figures and Tables

**Table 1:** Weekly progression of the 12-week running programme.

Week	Mon	Wed	Fri	Recovery	Intensity
1	10 × 1 min	30 min brisk walk	10 × 1 min	1.5 min	Run: Z3; Walk: Z2
2	10 × 1.5 min	35 min brisk walk	10 × 2 min	1.5 min	Run: Z3; Walk: Z2
3	8 × 3 min	40 min brisk walk	6 × 4 min	1 min	Run: Z3; Walk: Z2
4	6 × 5 min	45 min brisk walk	5 × 6 min	1 min	Run: Z3; Walk: Z2
5	3 × 8 min	45 min brisk walk	3 × 9 min	2 min	Run: Z3; Walk: Z2
6	2 × 10 min	45 min brisk walk	2 × 12 min	2–3 min	Run: Z3; Walk: Z2
7	2 × 14 min	45 min brisk walk	2 × 15 min	2–3 min	Run: Z3; Walk: Z2
8	2 × 18 min	45 min brisk walk	2 × 18 min	2.5–3 min	Run: Z3; Walk: Z2
9	2 × 20 min	45 min brisk walk	2 × 20 min	2–3 min	Run: Z3; Walk: Z2
10	2 × 20 min	60 min brisk walk	2 × 20 min	2–3 min	Run: Z3; Walk: Z2
11	45 min continuous	60 min brisk walk	45 min continuous	—	Run: Z3; Walk: Z2
12	60 min Continuous	60 min brisk walk	60 min continuous	—	Run: Z3; Walk: Z2

Abbreviations: Mon, Monday; Wed, Wednesday; Fri, Friday; Z2, Zone 2; Z3, Zone 3.

**Table 2:** Comparison of baseline characteristics between participants who completed the intervention and those who withdrew before study completion.

Variable	Completed (Sum of Ranks)	Withdrawn (Sum of Ranks)	U	Z	*p*-Value
Age	433.50	269.50	149.50	0.46	0.64
Body mass (kg)	402.00	301.00	149.00	−0.47	0.631
Body fat (%)	405.50	297.50	152.50	−0.37	0.710
Visceral fat rating	397.00	306.00	144.00	−0.63	0.525
BMI (kg/m^2^)	387.00	316.00	134.00	−0.94	0.345
Total body water (%)	417.00	286.00	164.00	−0.01	0.987
Standing long jump (cm)	418.50	284.50	164.50	0.00	1.000
Forward-reach flexibility (cm)	416.50	286.50	163.50	−0.03	0.975
Estimated VO_2_max (mL·kg^−1^·min^−1^)	340.50	289.50	130.50	−0.63	0.526

Abbreviations: BMI, body mass index; Estimated VO_2_max, estimated maximal oxygen uptake; U, Mann–Whitney U statistic; Z, standardized test statistic. Values for the completed and withdrawn groups represent the sum of ranks. Statistical significance was set at *p* < 0.05.

**Table 3:** Comparison of baseline demographic characteristics between the control and experimental groups.

Characteristic	Category	Control (*n* = 42), *n* (%)	Experimental (*n* = 22), *n* (%)	Total (*n* = 64), *n* (%)	*p*-Value
Sex	Male	7 (16.7)	7 (31.8)	14 (21.9)	0.208
	Female	35 (83.3)	15 (68.2)	50 (78.1)	
Age	18–19 years	33 (78.6)	19 (86.4)	52 (81.3)	0.521
	≥20 years	9 (21.4)	3 (13.6)	12 (18.8)	

Abbreviations: *n*, number of participants; %, percentage. *p*-values were calculated using Fisher’s exact test.

**Table 4:** Comparison of baseline outcome variables between the control and experimental groups.

Variable	Control (Sum of Ranks)	Experimental (Sum of Ranks)	U	Z	*p*-Value
Body mass (kg)	1350.50	729.50	447.50	−0.19	0.84
Body fat (%)	1401.50	678.50	425.50	0.50	0.61
Visceral fat rating	1377.00	703.00	450.00	0.16	0.87
BMI (kg/m^2^)	1356.00	724.00	453.00	−0.12	0.90
Total body water (%)	1326.00	754.00	423.00	−0.54	0.58
Standing long jump (cm)	1017.00	694.00	351.00	−0.71	0.47
Forward-reach flexibility (cm)	1388.00	692.00	439.00	0.31	0.75
Estimated VO_2_max (mL·kg^−1^·min^−1^)	908.00	632.00	278.00	−1.25	0.21

Abbreviations: BMI, body mass index; VO_2_max, estimated maximal oxygen uptake; U, Mann–Whitney U statistic; Z, standardized test statistic. Values for the control and experimental groups are presented as the sum of ranks. Statistical significance was set at *p* < 0.05.

**Table 5:** Sensitivity analysis of unadjusted changes between the control and experimental groups using Welch’s *t*-test.

Variable	Control Δ (Mean ± SD)	Experimental Δ (Mean ± SD)	*t*-Value	df	*p*-Value	Effect Size (Cohen’s d)
Body mass (kg)	1.46 ± 2.32	−1.37 ± 3.18	3.69	33.06	<0.001	1.02
Body fat (%)	−0.24 ± 1.61	−2.30 ± 1.62	4.84	42.52	<0.001	1.28
Visceral fat rating	0.19 ± 0.51	−0.50 ± 0.91	3.29	27.92	0.003	0.94
BMI (kg/m^2^)	0.32 ± 0.77	−0.91 ± 1.14	4.55	31.25	<0.001	1.27
Total body water (%)	−0.21 ± 1.06	0.61 ± 1.93	−1.86	27.85	0.073	0.53
Standing long jump (cm)	2.27 ± 7.92	5.36 ± 10.22	−1.20	37.26	0.238	0.34
Forward-reach flexibility (cm)	−2.05 ± 5.43	3.36 ± 5.02	−3.96	46.12	<0.001	1.03
Estimated VO_2_max (mL·kg^−1^·min^−1^)	1.63 ± 2.45	4.08 ± 3.82	−2.58	28.11	0.015	0.76

Abbreviations: Δ, mean change from baseline to post-intervention; SD, standard deviation; df, degrees of freedom; BMI, body mass index; VO_2_max, estimated maximal oxygen uptake. Effect sizes are presented as the absolute values of Cohen’s d (0.2 = small effect, 0.5 = medium effect, 0.8 = large effect).

**Table 6:** Primary analysis of intervention effects using ANCOVA.

Variable	Group (*n*)	Baseline (Mean ± SD)	Post (Mean ± SD)	Change (Mean ± SD)	95% CI for Change	Adjusted Mean Difference [95% CI]	*p*-Value	Holm-Adjusted *p*-Value	Partial η^2^
Body mass (kg)	Control (42)	68.33 ± 15.82	69.79 ± 16.70	1.46 ± 2.32	[0.76; 2.16]	−2.87 [−4.30; −1.44]	<0.001	<0.001	0.21
	Experimental (22)	68.36 ± 13.51	66.99 ± 13.32	−1.37 ± 3.18	[−2.70; −0.04]				
Body fat (%)	Control (42)	28.48 ± 7.36	28.24 ± 7.71	−0.24 ± 1.61	[−0.72; 0.25]	−1.99 [−2.86; −1.12]	<0.001	<0.001	0.26
	Experimental (22)	26.77 ± 7.08	24.47 ± 7.19	−2.30 ± 1.62	[−2.98; −1.62]				
Visceral fat rating	Control (42)	2.76 ± 2.14	2.95 ± 2.29	0.19 ± 0.51	[0.04; 0.34]	−0.67 [−1.02; −0.32]	<0.001	0.002	0.19
	Experimental (22)	2.82 ± 2.70	2.32 ± 2.21	−0.50 ± 0.91	[−0.88; −0.12]				
BMI (kg/m^2^)	Control (42)	23.65 ± 4.61	23.97 ± 4.85	0.32 ± 0.77	[0.09; 0.55]	−1.24 [−1.73; −0.74]	<0.001	<0.001	0.29
	Experimental (22)	23.29 ± 3.89	22.38 ± 3.46	−0.91 ± 1.14	[−1.39; −0.43]				
Total body water (%)	Control (42)	49.38 ± 5.67	49.16 ± 5.72	−0.21 ± 1.06	[−0.54; 0.11]	0.87 [0.12; 1.61]	0.023	0.047	0.08
	Experimental (22)	50.47 ± 5.84	51.08 ± 5.25	0.61 ± 1.93	[−0.20; 1.41]				
Standing long jump (cm)	Control (33)	150.15 ± 30.88	152.42 ± 27.84	2.27 ± 7.92	[−0.43; 4.97]	3.69 [−1.04; 8.42]	0.124	0.124	0.05
	Experimental (22)	159.09 ± 31.80	164.45 ± 31.24	5.36 ± 10.22	[1.09; 9.63]				
Forward-reach flexibility (cm)	Control (41)	53.20 ± 10.60	51.15 ± 11.13	−2.05 ± 5.43	[−3.71; −0.39]	5.32 [2.47; 8.16]	<0.001	0.002	0.19
	Experimental (22)	51.95 ± 12.61	55.32 ± 12.17	3.36 ± 5.02	[1.27; 5.46]				
Estimated VO_2_max (mL·kg^−1^·min^−1^)	Control (35)	29.09 ± 3.13	30.71 ± 4.06	1.63 ± 2.45	[0.82; 2.44]	2.51 [0.74; 4.28]	0.006	0.019	0.14
	Experimental (20)	30.62 ± 4.28	34.70 ± 5.25	4.08 ± 3.82	[2.40; 5.76]				

Note: Sample sizes differ across outcome variables because not all participants completed every outcome assessment at both baseline and follow-up. Analyses were performed using complete cases separately for each outcome. Missing data were not imputed.

## Data Availability

The data presented in this study are available on request from the corresponding author. The data are not publicly available due to privacy and ethical restrictions.

## Abbreviations

The following abbreviations are used in this manuscript:

ANOVA	Analysis of Variance
BMI	Body Mass Index
DXA	Dual-Energy X-ray Absorptiometry
HRmax	Maximum Heart Rate
SD	Standard Deviation
VO_2_max	Estimated maximal oxygen uptake

## References

[1] Castro O., Bennie J., Vergeer I., Bosselut G., Biddle S.J.H. (2020). How Sedentary Are University Students? A Systematic Review and Meta-Analysis. Prev. Sci..

[2] Jurakić D., Pedisić Z., Andrijasević M. (2009). Physical activity of Croatian population: Cross-sectional study using International Physical Activity Questionnaire. Croat. Med. J..

[3] Verma A.K., Singh G., Patwardhan K. (2022). Patterns of Physical Activity Among University Students and Their Perceptions About the Curricular Content Concerned With Health: Cross-sectional Study. JMIRx Med..

[4] Gropper S.S., Simmons K.P., Connell L.J., Ulrich P.V. (2012). Changes in body weight, composition, and shape: A 4-year study of college students. Appl. Physiol. Nutr. Metab..

[5] Lu Y., Wiltshire H.D., Baker J.S., Wang Q. (2021). The Effects of Running Compared with Functional High-Intensity Interval Training on Body Composition and Aerobic Fitness in Female University Students. Int. J. Environ. Res. Public Health.

[6] Swift D.L., Johannsen N.M., Lavie C.J., Earnest C.P., Church T.S. (2014). The role of exercise and physical activity in weight loss and maintenance. Prog. Cardiovasc. Dis..

[7] Willis L.H., Slentz C.A., Bateman L.A., Shields A.T., Piner L.W., Bales C.W., Houmard J.A., Kraus W.E. (2012). Effects of aerobic and/or resistance training on body mass and fat mass in overweight or obese adults. J. Appl. Physiol..

[8] American College of Sports Medicine (2021). ACSM’s Guidelines for Exercise Testing and Prescription.

[9] Scribbans T.D., Vecsey S., Hankinson P.B., Foster W.S., Gurd B.J. (2016). The Effect of Training Intensity on VO_2_max in Young Healthy Adults: A Meta-Regression and Meta-Analysis. Int. J. Exerc. Sci..

[10] Lowe A., Littlewood C., McLean S., Kilner K. (2017). Physiotherapy and physical activity: A cross-sectional survey exploring physical activity promotion, knowledge of physical activity guidelines and the physical activity habits of UK physiotherapists. BMJ Open Sport Exerc. Med..

[11] Blake H., Stanulewicz N., Mcgill F. (2017). Predictors of physical activity and barriers to exercise in nursing and medical students. J. Adv. Nurs..

[12] Ross R., Blair S.N., Arena R., Church T.S., Després J.P., Franklin B.A., Haskell W.L., Kaminsky L.A., Levine B.D., Lavie C.J. (2016). Importance of Assessing Cardiorespiratory Fitness in Clinical Practice: A Case for Fitness as a Clinical Vital Sign: A Scientific Statement From the American Heart Association. Circulation.

[13] Vračan D., Pisačić T., Slačanac K., Boris N. (2009). Stavovi prema vježbanju i interesi prema pojedinim sportskim aktivnostima studenata Arhitektonskog i Geodetskog fakulteta. Zbornik Radova 18. Ljetne Škole Kineziologa Republike Hrvatske.

[14] Foster C., Porcari J.P., Anderson J., Paulson M., Smaczny D., Webber H., Doberstein S.T., Udermann B. (2008). The Talk Test as a marker of exercise training intensity. J. Cardiopulm. Rehabil. Prev..

[15] Marín-Jiménez N., Perez-Bey A., Cruz-Leon C., Conde-Caveda J., Segura-Jimenez V., Castro-Piñero J., Cuenca-Garcia M. (2024). Criterion-related validity and reliability of the standing long jump test in adults: The Adult-Fit project. Eur. J. Sport Sci..

[16] Neljak B., Novak D., Sporiš G., Višković S., Markuš D. (2011). Methodology for Evaluating the Kinanthropological Characteristics of Students in Physical Education.

[17] Mayorga-Vega D., Aguilar-Soto P., Viciana J. (2015). Criterion-related validity of the 20-m shuttle run test for estimating cardiorespiratory fitness: A meta-analysis. J. Sports Sci. Med..

[18] Léger L.A., Mercier D., Gadoury C., Lambert J. (1988). The multistage 20 metre shuttle run test for aerobic fitness. J. Sports Sci..

[19] Bellicha A., van Baak M.A., Battista F., Beaulieu K., Blundell J.E., Busetto L., Carraca E.V., Dicker D., Encantado J., Ermolao A. (2021). Effect of Exercise Training on Weight Loss, Body Composition Changes, and Weight Maintenance in Adults with Overweight or Obesity: An Overview of 12 Systematic Reviews and 149 Studies. Obes. Rev..

[20] Bassett D.R., Howley E.T. (2000). Limiting Factors for Maximum Oxygen Uptake and Determinants of Endurance Performance. Med. Sci. Sports Exerc..

[21] Afonso J., Ramirez-Campillo R., Moscão J., Rocha T., Zacca R., Martins A., Milheiro A.A., Ferreira J., Sarmento H., Clemente F.M. (2021). Strength Training versus Stretching for Improving Range of Motion: A Systematic Review and Meta-Analysis. Healthcare.

[22] Behm D.G., Sale D.G. (1993). Velocity Specificity of Resistance Training. Sports Med..

